# Supplementary material for: Research on furniture image classification based on MobileNetNAK
Source: PeerJ Comput Sci. 2025 Sep 15;11:e3178. doi: 10.7717/peerj-cs.3178 (PMC12453834; doi:10.7717/peerj-cs.3178)
Supplement: Supplemental Information 3 [file peerj-cs-11-3178-s003.docx]

Data Availability Statement: Dataset for furniture image classification:

https://figshare.com/articles/dataset/archive/28831787?file=53855351

The furniture image classification task in this study involves two datasets. Dataset 1 includes furniture of varying ages, categorized into three classes as shown in Figure 6: (a) nearlyNewFurniture, with 317 images; (b) newFurniture, with 325 images; and (c) oldFurniture, with 303 images. The new furniture images were primarily sourced from brand-new furniture stores and online retailers, with the selected images featuring furniture without any signs of use. The nearly new furniture images were collected from the second-hand market, focusing on items with a freshness rating of over 90%, showing slight signs of use but no visible damage. The old furniture images were obtained from waste disposal stations and second-hand furniture markets, showcasing items with significant signs of use and visible damage. Dataset 2 is the Bonn Furniture Style dataset^[39],^ as shown in Figure 6(d), which includes 6 types of furniture: bed, chair, vanity, lamp, sofa, and table, and encompasses 17 different furniture styles. For the experimental dataset, approximately 1,000 images were randomly selected from each of the 17 styles, resulting in a total of 6,592 images.

The two datasets were divided into training, testing, and validation sets in an 8:1:1 ratio, with the validation set used during training and the testing set for model evaluation. The datasets exhibit a relatively balanced distribution of samples across categories, and the images cover a wide variety of furniture styles and designs. This ensures stability and fairness during the training and evaluation process, while also providing a rich and reliable data foundation for model validation experiments.

| 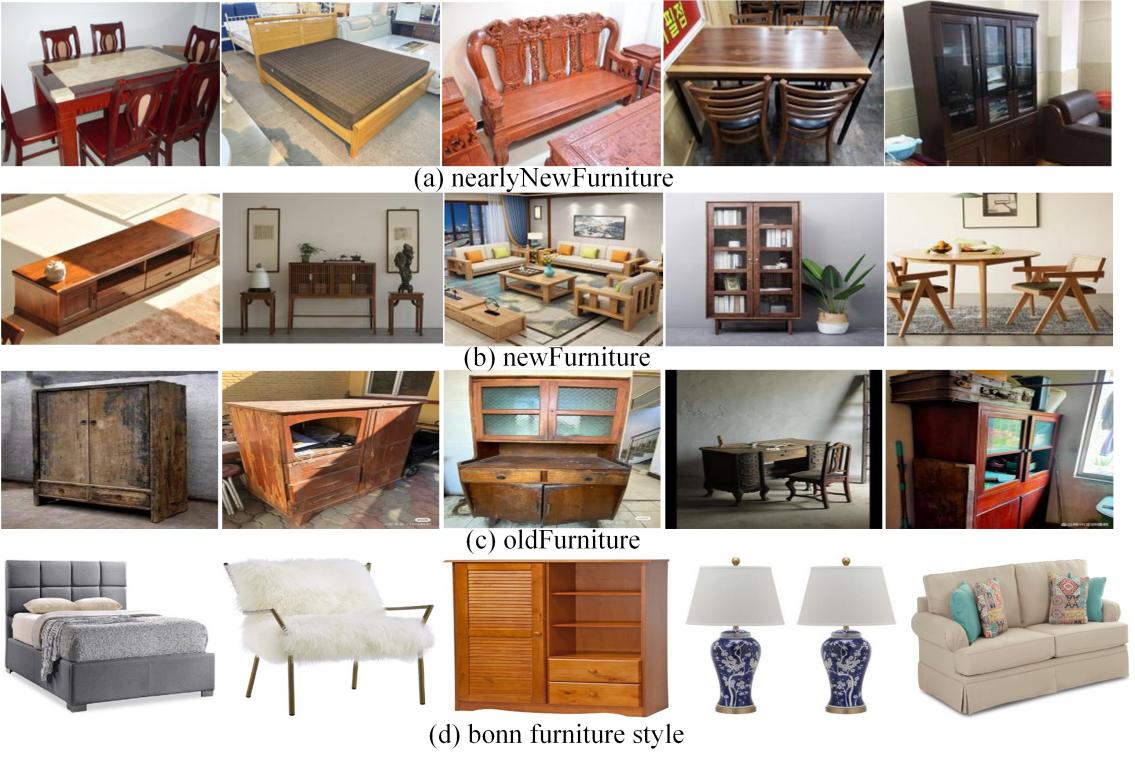 |
| --- |
| Figure 6. Data set example diagram. |
